# Targeting Several CAG Expansion Diseases by a Single Antisense Oligonucleotide

**DOI:** 10.1371/journal.pone.0024308

**Published:** 2011-09-01

**Authors:** Melvin M. Evers, Barry A. Pepers, Judith C. T. van Deutekom, Susan A. M. Mulders, Johan T. den Dunnen, Annemieke Aartsma-Rus, Gert-Jan B. van Ommen, Willeke M. C. van Roon-Mom

**Affiliations:** 1 Center for Human and Clinical Genetics, Leiden University Medical Center, Leiden, The Netherlands; 2 Prosensa Therapeutics B.V., Leiden, The Netherlands; 3 Leiden Genome Technology Center, Leiden University Medical Center, Leiden, The Netherlands; University of Pittsburgh School of Medicine, United States of America

## Abstract

To date there are 9 known diseases caused by an expanded polyglutamine repeat, with the most prevalent being Huntington's disease. Huntington's disease is a progressive autosomal dominant neurodegenerative disorder for which currently no therapy is available. It is caused by a CAG repeat expansion in the *HTT* gene, which results in an expansion of a glutamine stretch at the N-terminal end of the huntingtin protein. This polyglutamine expansion plays a central role in the disease and results in the accumulation of cytoplasmic and nuclear aggregates. Here, we make use of modified 2′-*O*-methyl phosphorothioate (CUG)n triplet-repeat antisense oligonucleotides to effectively reduce mutant huntingtin transcript and protein levels in patient-derived Huntington's disease fibroblasts and lymphoblasts. The most effective antisense oligonucleotide, (CUG)_7_, also reduced mutant ataxin-1 and ataxin-3 mRNA levels in spinocerebellar ataxia 1 and 3, respectively, and atrophin-1 in dentatorubral-pallidoluysian atrophy patient derived fibroblasts. This antisense oligonucleotide is not only a promising therapeutic tool to reduce mutant huntingtin levels in Huntington's disease but our results in spinocerebellar ataxia and dentatorubral-pallidoluysian atrophy cells suggest that this could also be applicable to other polyglutamine expansion disorders as well.

## Introduction

Polyglutamine (polyQ) diseases are a group of disorders caused by CAG triplet repeat expansions in the coding region of the genome. The disease causing proteins in these polyQ diseases are very different, but in each case the expanded stretch of glutamines results in a toxic-gain-of function of the protein and this leads to neurodegeneration. To date, a total of 9 polyQ disorders have been described: dentatorubral-pallidoluysian atrophy (DRPLA), Huntington's disease (HD), spinal bulbar muscular atrophy (SBMA), and spinocerebellar ataxias (SCA1, 2, 3, 6, 7, and 17) [1], [2]. Of these polyQ disorders, HD and SCA3 have the highest prevalence worldwide [3]. The expanded repeats in these polyQ diseases are unstable resulting in anticipation; a more severe and earlier onset of disease in following generations [4]. There is an inverse correlation of disease onset and polyQ length in the protein; the longer the CAG repeat, the earlier the age of onset of the disease [1]. Protein aggregates are found in the nucleus and cytoplasm of cells, indicating that protein misfolding is a common feature of these disorders. Currently no treatment is available to delay onset or even slow progression of polyQ diseases.

In HD, the expanded CAG repeat is located in the first exon of the *HTT* gene on chromosome 4p16. The expanded CAG transcript is translated into a mutant huntingtin (htt) protein with an expanded polyQ tract at the N-terminus. Patients with 39 or more CAG repeats will develop the disease, whereas people with 35 to 38 repeats show reduced penetrance [5]. The disease is characterized by motor, psychiatric and cognitive impairments and the typical age of onset lies between 30 and 50 years [6]. The major neuropathology occurs in the striatum but degeneration is seen throughout the brain when the disease progresses. Various other proteins have been found to co-localize with htt aggregates, i.e. TATA box binding protein (TBP), CREB binding protein (CBP) and several molecular chaperones [7]–[10]. When the mutation for HD was found, htt was a protein of unknown function but extensive research over the past decade has revealed numerous functions for htt. Also many affected cellular processes have been identified in HD, such as transcriptional de-regulation, mitochondrial dysfunction, and impaired vesicle transport [3], [11].

SCAs are genetically and clinically distinct autosomal dominant CAG-expansion diseases, numbered by the order of gene description. Patients with SCA exhibit cerebellar degeneration resulting in ataxia and oculomotor deficits, often followed by general brain atrophy [12], [13]. The first SCA identified, SCA1, is caused by a CAG repeat expansion of 41 or more in exon 8 of the *ATXN1* gene [3]. *ATXN1* is translated into the 98 kDa protein ataxin-1, which is involved in transcriptional regulation and RNA metabolism [14]. Mutated ataxin-1, by entering the nucleus, causes cellular dysfunction [15]. In SCA3, the expanded CAG repeat is located in exon 10 of the *ATXN3* gene which is translated into mutant ataxin-3 [16]. Patients develop the disease when the number of CAGs exceed 51, while there is reduced penetrance when the number of repeats is between 45 and 51 [17]. The 42 kDa ataxin-3 protein is suggested to be involved in proteasomal degradation and transport of ubiquitinated proteins [18]. DRPLA is a rare autosomal dominant disorder, characterized by dementia, ataxia, chorea, myoclonic epilepsy, and psychiatric disturbances. The disease is caused by a CAG repeat expansion in exon 5 of the *ATN1* gene, which encodes the 200 kDa atrophin-1 protein. Atrophin-1 is a known transcriptional co-regulator although its exact function is not well understood [19]. Patients with a repeat of 49 or more glutamines will develop the disease [20].

Most therapeutic strategies under investigation for polyQ disorders are aimed at counteracting one of the many cellular processes that are altered due to expression of the mutant protein. For instance, in all of these neurodegenerative diseases the formation of fragmented protein products by proteolytic cleavage is an important step in the pathogenic process [3]. It has been shown that altering proteolysis of the mutant htt protein can be beneficial, as an HD mouse model lacking the caspase 6 cleavage site had reduced neuronal dysfunction and neurodegeneration [21]. Reducing mutant polyQ protein levels and thereby inhibiting all downstream toxic effects would be much more effective than targeting a single cellular process. One way to achieve this would be to enhance the degradation of mutant polyQ proteins through activation of the proteasome [22] or through upregulation of the autophagic pathway [23]. Another strategy would be to inhibit the formation of mutant polyQ proteins by gene silencing or transcript degradation [24]. RNAi is increasingly used as a potential therapeutic tool to reduce expression of target transcripts [25]. RNAi is an endogenous cellular defense mechanism against exogenous viral components and is also involved in transcriptional regulation [26]. Specific knock down of target sequences is achieved by introducing exogenously modified oligonucleotides (e.g. short hairpin RNA (shRNA) and short interfering RNA (siRNA)) that bind to the target transcript, which is subsequently degraded or its translation blocked. Recently an siRNA targeting both normal and mutant htt was found to be well-tolerated in wild-type rats [27]. However, endogenous htt expression is important for normal cellular function, as underlined by the finding that conditional knockout of murine htt in forebrain and testis resulted in loss of function and progressive neurodegeneration [28]. Total loss of the endogenous htt homolog in a Drosophila HD model expressing the human first exon of the *HTT* gene with 93 Qs enhanced the HD pathogenesis [29]. These studies show that a specific reduction of mutant htt levels, leaving as much wild type htt protein as possible, would be the optimal outcome of a therapy aimed at htt knockdown. Specific reduction of the mutant htt transcript was shown by allele-specific siRNAs directed against a single nucleotide polymorphism (SNP) in htt exon 50 [30]. In a recent study on the cleavage of triplet repeat hairpins by ribonuclease dicer it was shown that an siRNA with 7 consecutive CUG nucleotides specifically reduced the expression of the mutant htt transcript containing 44 CAG repeats in HD human fibroblasts [31]. Although off-target effects and interference with endogenous RNAi processes remains to be assessed [32], these results are encouraging.

Another RNA based therapy approach to knock down gene or protein expression is the use of single stranded antisense oligonucleotides (AONs). One of the most promising examples of AON treatment in a neurodegenerative disease is aimed at amyotrophic lateral sclerosis (ALS). In ∼2% of ALS patients, the disease is caused by a mutation in Superoxide dismutase 1 (SOD1) [33]. Continuous intraventricular infusion of AONs successfully down regulated SOD1 mRNA and protein levels in the brain and significantly slowed disease progression in an animal model of ALS [34]. A clinical trial is currently ongoing in ALS patients with SOD1 mutations and results are expected this year [35].

For glutamine-expansion disorders, peptide nucleic acid (PNA) and locked nucleic acid (LNA) antisense oligomers targeting CAG repeats have been used to reduce expanded HD and SCA3 transcripts *in vitro* [36]–[39]. However, although PNA transfection efficiently reduced mutant protein levels with very long glutamine expansions, the reductions on polyQ lengths that occur most frequently in the HD patient population were less pronounced [38], [39]. In the current we make use of 2′-*O*-methyl (2′OMe) modified RNA AONs with a phosphorothioate (PS) backbone carrying different CUG numbers. We examine the effect of (CUG)_n_ AONs on mRNA level in cell lines derived from HD, SCA1, SCA3, and DRPLA patients with CAG expansions that occur most frequently in the patient population. A significant reduction in expanded transcript levels was found in patient derived fibroblast from HD, SCA1, SCA3, and DRPLA. Furthermore a significant reduction of mutant htt protein was seen in the HD cells. For htt, a reduction in wild-type htt transcript levels was observed as well, but this reduction was less pronounced than for the mutant transcript. Lowering the AON concentration increased the specificity for the mutant transcript. These results show that one single antisense oligonucleotide could be a promising therapeutic treatment for all polyQ disorders.

## Results

### (CUG)_7_ AON shows most pronounced reduction of HTT transcript levels

**Figure 1:**
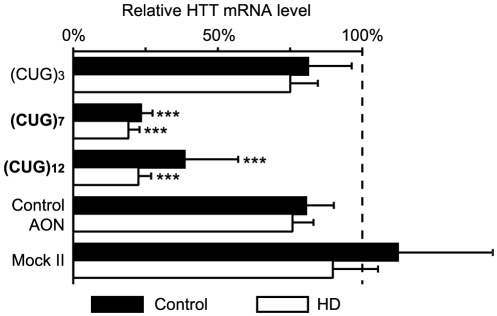
Number of CUGs of AON influences the reduction of HTT transcript levels. Total RNA was isolated 48 hours after transfection. Quantitative RT-PCR was used to measure HTT mRNA levels in control and HD fibroblasts after treatment with 100 nM (CUG)_3_, (CUG)_7_, (CUG)_12_ AON, 100 nM non-htt specific h40AON2 (Control AON), transfection agent only (Mock II), or non-transfected cells (Mock I, not included in this figure). ACTB and RPL22 are used as reference genes. The expression level of Mock I transfections are set to 100%. For all transfections n = 6 and *** *P*<0.001).

Patient-derived human fibroblasts were transfected with AONs with 3, 7 and 12 consecutive CUGs ((CUG)_3_, (CUG)_7_, and (CUG)_12_, respectively) and total RNA was isolated after 48 hours. In the *HTT* gene the glutamine repeat consists of a CAG stretch, followed by one CAA and a final CAG triplet. The HD cell line GM04022 contained a (CAG)*_n_* CAA CAG repeat with *n* = 18 and 44. As a control fibroblasts cell line FLB73 was used where *n* = 17 and 21. To avoid influences of CAG repeat length, reductions in total HTT mRNA levels were measured by quantitative PCR (qPCR) with primers within the CAG containing exon but amplifying a transcript fragment upstream of the repeat **(Table S1)**. The most significant reduction in total HTT transcript of 81% (±4%) in the HD and 76% (±4%) in the control fibroblasts was found after (CUG)_7_ treatment **(** **Fig. 1** **)**. (CUG)_12_ transfection resulted in a significant reduction of total HTT transcript of 78% (±5%) in the HD and 61% (±18%) in the control cell line. The (CUG)_3_ did not show significant reduction of HTT mRNA levels. The (CUG)_7_ AON was selected for further testing since it was the shortest AON resulting in the most significant reduction in HTT mRNA levels.

### Reduction of mutant HTT mRNA levels in HD cells after (CUG)_ 7_ treatment

**Figure 2:**
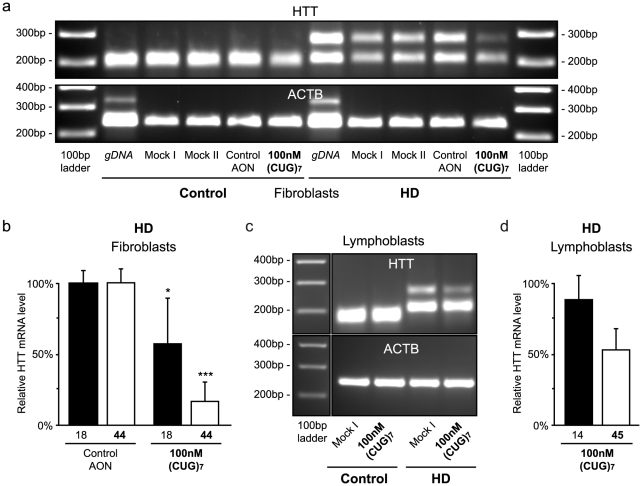
Effect of (CUG)_7_ AON on HTT mRNA levels in HD patient derived cell lines 48 hours after transfection. Cells were transfected with 100 nM (CUG)_7_, non-htt specific h40AON2 (Control AON), transfection agent only (Mock II), or non-transfected cells (Mock I). **(a)** Agarose gel analysis of the HTT transcript with primers flanking the CAG repeat of control (FLB73) and HD (GM04022) fibroblasts treated with various AONs. Transfection with (CUG)_7_ shows a decrease of the upper band, representing the transcript from the mutant allele. The lower band, representing the normal HTT transcript, is also reduced, but to a lesser extent. Control cells treated with (CUG)_7_ only show a slight reduction compared to the control transfections. PCR products with primers for ACTB were used as loading control. gDNA was taken along to control for the PCR reaction over the CAG repeat. **(b)** Lab-on-a-Chip analysis of HTT transcripts after (CUG)_7_ treatment in a HD fibroblast cell line. The mutant transcript, with 44 CAGs, is significantly reduced by 83% after (CUG)_7_ treatment, compared to transfection controls. The normal HTT transcript with 18 CAGs is reduced by 43%. Expression levels are corrected for loading differences with ACTB. The mRNA level of the Mock I transfection was set on 100% (* *P*<0.05, *** *P*<0.001, n = 4). **(c)** Agarose gel analysis of HTT transcripts after (CUG)_7_ treatment in EBV transformed control and HD human lymphoblasts. After transfection with (CUG)_7_ the mutant HTT transcript with 45 CAGs is decreased compared to the Mock transfection. No changes in intensity of the HTT transcripts from the control lymphoblasts are seen after (CUG)_7_ treatment. **(d)** Lab-on-a-Chip analysis of HTT transcripts after PS57 treatment of human HD lymphoblasts. Mutant HTT transcript is reduced by 46% after (CUG)_7_ treatment, whereas the normal HTT allele shows an 11% reduction. (n = 2)

Since regular htt expression is important for normal cellular function, our approach is to lower mutant htt protein levels, while maintaining sufficient levels of normal protein. To examine the effect of (CUG)_7_ treatment on both HTT transcripts an allele-specific PCR with primers flanking the CAG repeat was performed in quadruplo **(** **Fig. 2a** **)**. The mutant transcript was decreased by 83% (±13%, measured by Lab-on-a-Chip analysis) in (CUG)_7_ treated cells compared to controls, while normal transcript was reduced to a lesser extent with 43% (±32%) (**Fig. 2b** **)**. Treatment of the control cell line with (CUG)_7_ showed a reduction for both alleles of 21% (±38%) and 40% (±38%) respectively.

We repeated this experiment in duplo in patient-derived Epstein Barr Virus transformed control and HD lymphoblasts **(** **Fig. 2c** **)**. (CUG)_7_ transfection of the HD cell line gave a reduction of the mutant transcript of 53% (±10%), while only a small decrease of 22% (±11%) for the normal transcript was detected **(** **Fig. 2d** **)**. No apparent reduction in the control cell line was found (data not shown).

### Reduction of mutant htt protein levels in a HD cell line after (CUG)_7_ treatment

**Figure 3:**
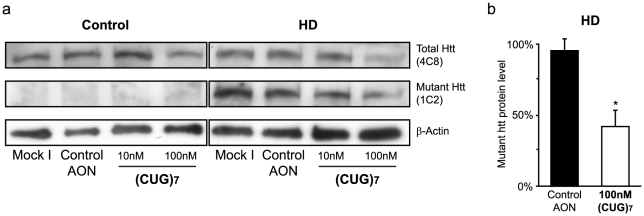
(CUG)_7_ AON reduces mutant htt protein levels in HD patient fibroblast cell lines. Cells were transfected with 10 nM and 100 nM (CUG)_7_, non-htt specific h40AON2 (Control AON), or non-transfected cells (Mock I). **(a)** Western blot of control (FLB73) and HD (GM04022) fibroblasts treated with (CUG)_7_ and controls. Total (4C8) and mutant (1C2) htt protein expression is reduced 72 hours after treatment with (CUG)_7_. No mutant htt could be detected in the control fibroblasts with 1C2. β-actin is used as loading control. **(b)** Mutant htt protein levels in HD (GM04022) fibroblasts after 100 nM (CUG)_7_ transfection were quantified by ImageJ software. A significant reduction of 58% of mutant htt protein was seen after (CUG)_7_ transfection as compared to control transfections (* *P*<0.05, n = 2). Mutant protein levels of Mock I transfection were set to 100%.

Since mRNA levels of the HTT transcript were substantially reduced after treatment with (CUG)_7_, in both experiments, we investigated htt protein levels **(** **Fig. 3a** **)**. Antibody 4C8 can be used to detect total htt protein [40], while antibody 1C2 specifically recognizes the expanded polyQ tract [41]. Patient-derived human fibroblasts were transfected and protein isolated (see Materials and Methods). 96 hours after first treatment of HD fibroblasts with 100 nM (CUG)_7_ 4C8 antibody showed a clear reduction of 54% (±34%) in htt protein level, while a less pronounced reduction of 16% (±28%) was observed in the control fibroblasts **(** **Fig. 3a** **and data not shown)**. With 1C2 antibody a significant reduction of 58% (±16%) of mutant htt protein was seen in the HD fibroblasts following 100 nM (CUG)_7_ treatment **(** **Fig. 3b** **)**. Thus, reduction of mutant htt protein was more pronounced than normal htt.

### (CUG)_7_ AON efficiency is concentration dependent

**Figure 4:**
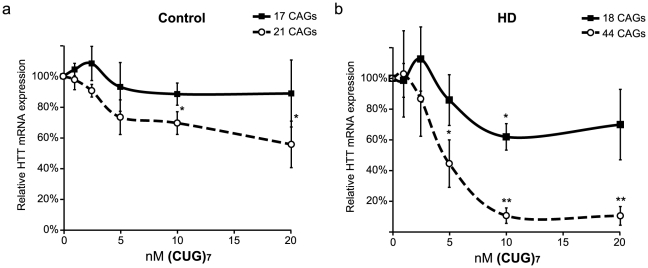
Effect of various (CUG)_7_ AON concentrations on HTT mRNA expression. Cells were transfected with 1–20 nM (CUG)_7_. PCR products with primers flanking the CAG repeat of HTT were quantified by Lab on a Chip. **(a)** In the control cell line (FLB73) both alleles (17 and 21 CAGs) show a comparable concentration dependent reduction of HTT mRNA quantification after (CUG)_7_ transfection. **(b)** In HD fibroblasts (GM04022) the mutant transcript, with 44 CAGs, shows a strong reduction of mutant HTT mRNA expression with increasing (CUG)_7_ AON concentrations, whereas the normal HTT transcript with 18 CAGs is reduced to a lesser degree. Expression levels are corrected for loading differences with ACTB and mRNA levels of the Mock I transfections were set on 100% (* *P*<0.05, ** *P*<0.01, n = 4).

To test if (CUG)_7_ AON concentration is related to efficacy, various AON concentrations were used to transfect HD and control fibroblasts. Lab-on-a-Chip analysis **(** **Fig. 4a and b** **)** showed a reduction of mutant HTT with an IC_50_ value between 2.5 nM and 5 nM **(** **Fig 4b** **)**. At 10 nM (CUG)_7_ the mRNA expression of mutant HTT was reduced by 89% (±5%), whereas normal HTT transcript was reduced by 38% (±9%) in the HD fibroblasts. HTT mRNA reduction was less pronounced for both alleles (16% (±6%) and 36% (±5%)) in the control cells, suggesting that at lower concentrations the (CUG)_7_ AON is more specific at reducing HTT transcripts with expanded CAG repeats **(** **Fig 4a** **)**.

### AON directed against the CAG repeat reduces mutant ataxin-3 levels

**Figure 5:**
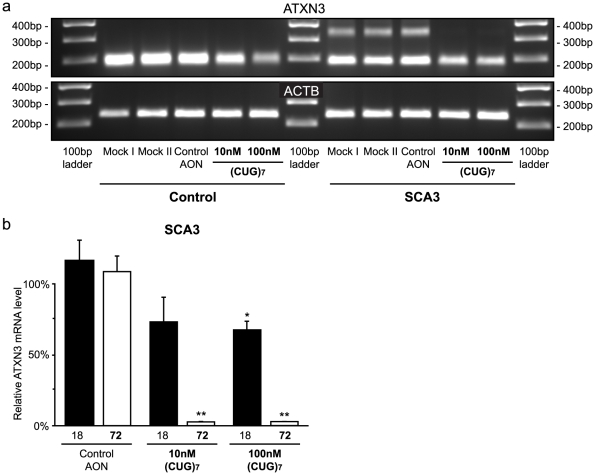
(CUG)_7_ AON reduces mutant ATXN3 mRNA expression in patient-derived fibroblasts. Cells were transfected with 10 or 100 nM (CUG)_7_, non-htt specific h40AON2 (Control AON), transfection agent only (Mock II), or non-transfected cells (Mock I). **(a)** Agarose gel analysis with primers flanking the CAG repeat in the ATXN3 transcript of control (FLB73) and SCA3 (GM06151) fibroblasts after (CUG)_7_ treatment. After transfection with (CUG)_7_ the upper band, representing the mutant ATXN3 transcript, is greatly decreased in intensity, while the lower band, representing the wild-type transcript, is only slightly reduced. β-actin was used as loading control. **(b)** Lab-on-a-Chip analysis of ATXN3 transcripts after 10 nM and 100 nM (CUG)_7_ treatment in a SCA3 (GM06151) fibroblast cell line. The mutant transcript, with 72 CAGs, is significantly reduced by 97% after (CUG)_7_ treatment, compared to transfection controls. The normal ATXN3 transcript with 18 CAGs is reduced by 27% and 33% after 10 nM and 100 nM (CUG)_7_ AON treatment, respectively. Expression levels are corrected for loading differences with β-actin. The mRNA level of the Mock I transfection was set on 100% (* *P*<0.05, ** *P*<0.01, n = 2).

Since CAG repeat expansions are a hallmark of several neurodegenerative disorders, we tested the molecular efficacy of our AON approach to reduce the expression of other genes as well. SCA3 patients have a CAG triplet repeat expansion in the *ATXN3* gene, we examined the effect of (CUG)_7_ treatment in patient-derived SCA3 fibroblasts with a CAG CAA (CAG)*_n_* repeat where *n* = 18 and 72. As for htt, the (CUG)_7_ treatment reduced the transcript from the expanded ataxin-3 allele, while reduction in transcript levels from the normal allele was less pronounced **(** **Fig. 5a** **)**.

PCR with primers amplifying a product containing the CAG repeat in ATXN3 showed a significant 97% (±1%) down regulation of mutant ATXN3 after both 10 nM and 100 nM (CUG)_7_ AON transfection **(** **Fig. 5b** **)**. The wild type allele was reduced by respectively 27% (±17%) and 33% (±6%) by 10 nM and 100 nM after (CUG)_7_ AON treatment.

### Reduction on other expanded CAG transcripts by (CUG)_7_ treatment

**Figure 6:**
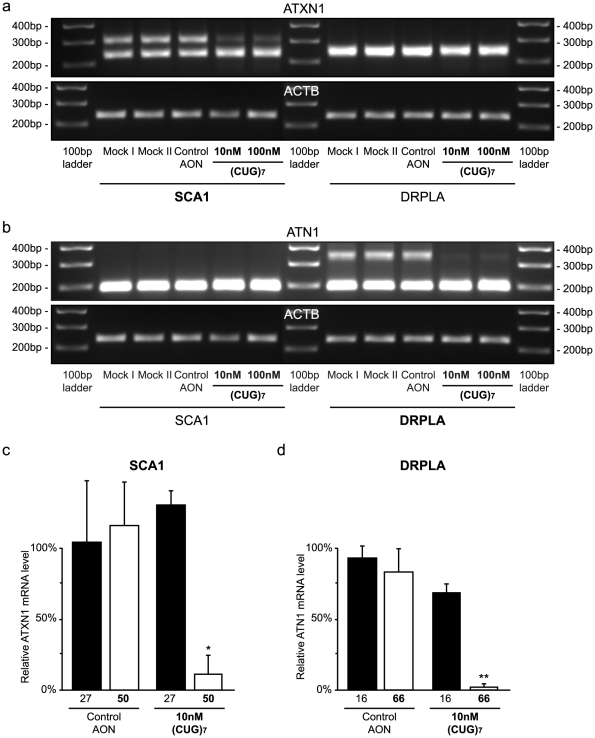
(CUG)_7_ AON reduces mutant ATXN1 and ATN1 transcripts in SCA1 and DRPLA fibroblasts. SCA1 (GM06927) and DRPLA (GM13716) patient derived fibroblasts were transfected with 10 and 100 nM (CUG)_7_, 10 nM non-htt specific h40 AON2 (Control AON), transfection agent only (Mock II), or non-transfected cells (Mock I). **(a)** Agarose gel analysis with primers flanking the CAG repeat in the ATXN1 transcript. After transfection with both 10 nm and 100 nM (CUG)_7_ the upper band, representing the mutant ATXN1 transcript, is greatly decreased in intensity, while the lower band, representing the wild-type transcript, is not reduced. β-actin was used as loading control. **(b)** Agarose gel analysis with primers flanking the CAG repeat in the ATN1 transcript. After transfection with both 10 nM and 100 nM (CUG)_7_, the upper band representing the mutant ATN1 transcript, is greatly decreased in intensity, while the lower band representing the wild-type transcript, is not reduced. β-actin was used as loading control. **(c)** Lab-on-a-Chip analysis of ATXN1 transcripts in SCA1 cells after control AON and 10 nM (CUG)_7_ treatment. The mutant transcript, with 72 CAGs, is significantly reduced by 89% after (CUG)_7_ treatment, compared to transfection controls. The normal ATXN1 transcript with 27 CAGs is not reduced. **(d)** ImageJ analysis of ATN1 transcripts in DRPLA cells after control AON and 10 nM (CUG)_7_ treatment. The 66 CAGs containing mutant ATN1 transcript is significantly reduced by 98% after (CUG)_7_ treatment, while normal ATN1 transcript with 16 CAGs is not significantly reduced by 30%. Expression levels are corrected for loading differences with β-actin. The mRNA level of the Mock I transfection was set on 100% (* *P*<0.05, ** *P*<0.01, n = 3).

We next tested SCA1 and DRPLA fibroblasts. Allele-specific PCRs with primers flanking the CAG repeat were performed to examine the effect of (CUG)_7_ treatment in both the normal and mutant allele. The mutant ataxin-1 (ATXN1) transcript was decreased by 89% (±14%) in 100 nM (CUG)_7_ treated SCA1 cells compared to control transfections **(** **Fig. 6a and c** **)**, while the normal transcript was not reduced. (The SCA1 and DRPLA cell lines served as each other's control.) Mutant atrophin-1 (ATN1) in DRPLA was also reduced after 100 nM (CUG)_7_ treatment by 98% (±2%), whereas there was only a 30% (±6%) reduction in the normal allele **(** **Fig. 6b and d** **)**.

### (CUG)_7_ does not affect other endogenous CAG-enclosing transcripts

The human genome contains several proteins that contain polyQ tracts, usually encoded by a combination of CAG and CAA triplets. Most of these transcripts are essential for normal cellular function [42] so reducing those transcripts could impair normal cellular function. To verify whether other uninterrupted CAG repeat containing transcripts were affected, 5 other transcripts were selected after a BLAST search: androgen receptor (AR), ataxin-2 (ATXN2), glutaminase (GLS), TBP, and zinc finger protein 384 (ZNF384). For the cells used in the present study the exact CAG tract length of these 5 transcripts was first determined by Sanger sequencing **(** **Table 1** **)**. Primers for qPCR were designed within the CAG containing exon but amplifying a fragment downstream of the CAG repeat in the transcript **(Table S1)**. For technical reasons primers for ATXN2 were designed upstream of the CAG repeat.

**Table 1:** Number of uninterrupted CAGs and codons that encode for glutamine in CAG repeat enclosing transcripts as determined by Sanger sequencing and a summary of the effect of (CUG)_7_ treatment in those transcripts.

Transcript Name	Cell Line	Glutamine stretch		Uninterrupted CAGs		Significant reduction after 100 nM (CUG)_7_ AON
		Allele1	Allele2	Allele 1	Allele 2	
AR	Control	22	24	21	23	No
	HD	23	24	22	23	No
**ATN1**	Control	19	20	15	16	No
	HD	12	19	8	15	No
	**DRPLA**	**20**	**70**	**16**	**66**	**Yes**
	SCA1	20	20	16	16	No
**ATXN1**	DRPLA	29	31	14	15	No
	**SCA1**	**29**	**52**	**14**	**37**	**Yes**
ATXN2	Control	20	20	8	8	No
	HD	20	20	8	8	No
**ATXN3**	**Control**	**17**	**19**	**15**	**17**	**Yes**
	HD	19	23	17	21	No
	**SCA3**	**20**	**74**	**18**	**72**	**Yes**
GLS	Control	8	14	8	14	No
	HD	7	18	7	18	No
**HTT**	**Control**	**19**	**23**	**17**	**21**	**Yes**
	**HD**	**20**	**46**	**18**	**44**	**Yes**
TBP	Control	37	38	17	18	No
	HD	35	36	16	17	No
ZNF384	Control	15	16	14	15	No
	HD	15	16	14	15	No

*Abbreviations*: AR, androgen receptor; ATN1, atrophin-1; ATXN1, ataxin-1; ATXN2, ataxin-2; ATXN3, ataxin-3; GLS, glutaminase; HTT, huntingtin; TBP, TATA box binding protein; ZNF384, zinc finger protein 384. Reduced transcripts after (CUG)_7_ treatment are depicted in bold.

**Figure 7:**
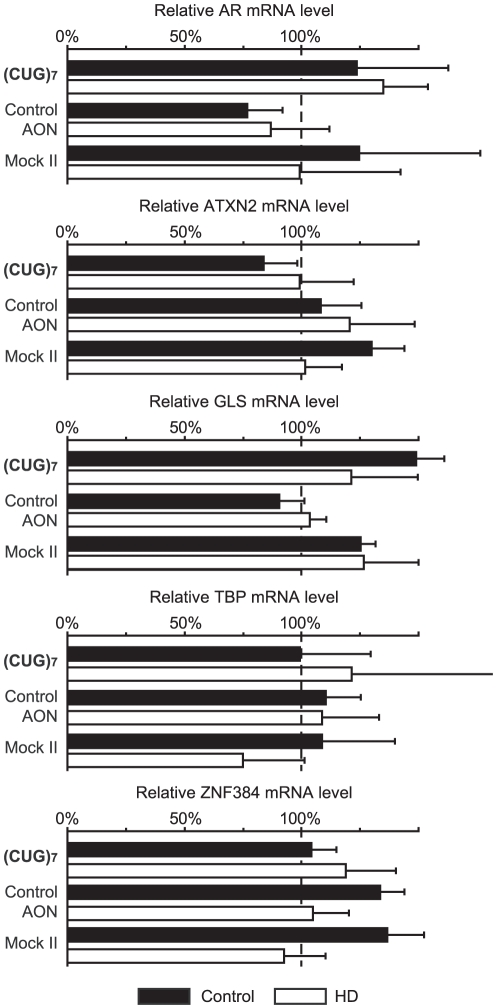
(CUG)_7_ AON does not affect other CAG-containing transcripts. Quantitative real-time PCR was used to measure androgen receptor (AR), ataxin-2 (ATXN2), glutaminase (GLS), TATA box binding protein (TBP), and zinc finger protein 384 (ZNF384) mRNA levels in control and HD fibroblasts after treatment with 100 nM (CUG)_7_, non-htt specific h40AON2 (Control AON), transfection agent only (Mock II), or non-transfected cells (Mock I). All tested CAG-enclosing transcripts were unaffected by (CUG)_7_ treatment. ACTB and RPL22 are used as reference genes. The expression level of Mock I transfections were set on 100% (n = 6).

**Figure 8:**
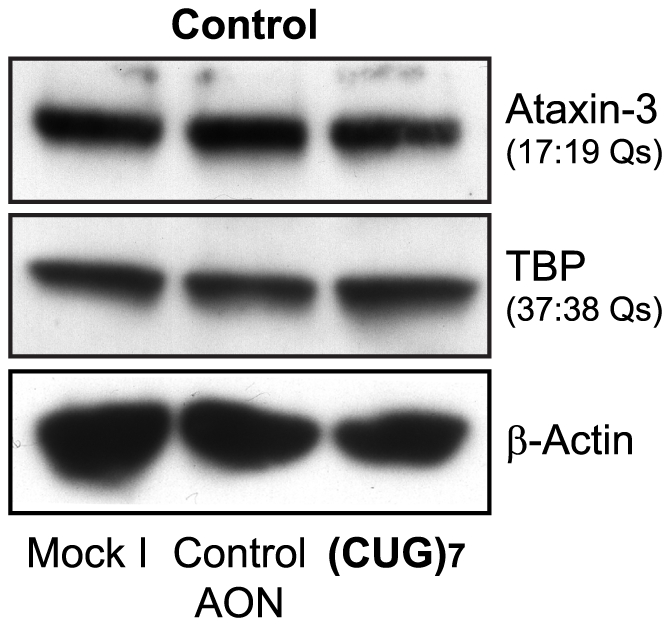
(CUG)_7_ AON does not reduce other polyQ-containing proteins. Western blot of control (FLB73) fibroblasts treated with 100 nM (CUG)_7_, non-htt specific h40AON2 (Control AON), and non-transfected (Mock I). TATA box binding protein (TBP) and ataxin-3 are not reduced 72 hours after treatment with (CUG)_7_. β-actin is used as loading control.

All tested CAG-enclosing transcripts were unaffected by 100 nM (CUG)_7_ treatment **(** **Fig. 7** **)**, including the AR transcript that contained CAG repeats of 21 and 23 CAGs. Endogenous ataxin-3 (with 17∶18 Qs) and TBP (37∶38 Qs) protein levels were unaffected by 100 nM (CUG)_7_ treatment **(** **Fig. 8** **)**. From the above results we can conclude that (CUG)_7_ does not significantly reduce endogenous CAG containing transcripts and does not decrease endogenous polyQ-containing protein levels.

## Discussion

The present study shows that an AON targeting CAG repeats and consisting of 7 CUGs significantly reduces protein and RNA levels of mutant htt in patient-derived fibroblast cell lines. This reduction was also seen, but to a lesser extends with (CUG)_12_ but not with (CUG)_3_. Although there was also a reduction of normal HTT transcript levels, the results show a preferential allele specific reduction of mutant HTT in patient derived HD cells and this allele specificity was improved when AON concentration was lowered from 100 nM to 10 nM.

Furthermore, other non-expanded CAG-containing transcripts that were investigated were not affected by (CUG)_7_ treatment. There was no reduction after (CUG)_7_ treatment of the AR transcript that contained the longest tested uninterrupted CAG repeat, namely 21 and 23 CAGs. Normal HTT that contained 17 and 21 CAG repeats did show a reduction after (CUG)_7_ treatment, suggesting that there are other factors besides the number of consecutive CAG triplets that determine (CUG)_7_ efficacy.

The results with mutant ATXN1, ATXN3, and ATN1 confirmed the specificity of (CUG)_7_ for transcripts with an expanded CAG tract in SCA1, 3, and DRPLA patient derived cells, respectively. Our results suggest that (CUG)_7_ could be effective in reducing expanded CAG repeat containing transcripts in all polyQ diseases.

In HD there is a gain of toxic function of the mutant htt protein, while regular htt expression is important for normal cellular function. Knockout of the homologous htt mouse gene was found to be early embryonic lethal [43] and previous studies have shown that approximately 50% of htt protein level is required to maintain cell functionality [28], [44]–[46]. In addition, increased clearance of mutant htt protein by autophagy in a *Drosophila* model and blockage of mutant htt in a conditional knock-out mouse model of HD resulted in a reduction in aggregates and an ameliorated phenotype [47], [48]. Reduction of mutant protein levels will therefore most likely result in amelioration of the toxic HD phenotype but total knockdown of htt protein expression would not be advantageous [49].

For other polyQ disorders the role of wild-type polyQ proteins in adult brain is still poorly understood. In a SCA3 *Drosophila* model expressing normal and mutant human ataxin-3, loss of normal ataxin-3 contributed to neurodegeneration [50]. In contrast, non allele-specific reduction of endogenous ataxin-3 was not found to be detrimental in rodents [51], [52]. Ataxin-1 knockout mice resulted in cerebellar transcriptional changes resembling SCA1 pathology, suggesting a neuroprotective role of normal ataxin-1 [53]. In contrast, atrophin-1 knockout mice were viable and did not show a clear phenotype [54], suggesting that non allele-specific reduction of both alleles in DRPLA is not harmful. Future research is necessary to determine the significance of wild-type polyQ protein levels for normal cellular function and the importance of AON-mediated allele-specific transcript reduction.

Several papers have shown allele-specific silencing of mutant htt with single nucleotide polymorphism (SNP)-specific siRNAs [30], [55]. Indeed HD patients carry different SNPs, requiring the development of at least five different siRNAs, to target 75% of the European and United States HD population [56], [57]. However, the advantage of the approach described in the current paper is that it requires only 1 AON to treat all HD patients and would be applicable in other polyQ diseases. Furthermore, siRNAs are double stranded oligonucleotides and these have been described to cause off-target effects by the sense strand, [58] as well as striatal toxicity [32], [59]. In addition, RNA interference is an endogenous process; addition of siRNAs might cause toxicity due to an overload of the endogenous system. Recently, nucleic acids conjugates, with different chemistries than the AONs used in the current study, were used for allele-specific silencing of mutant htt. PNAs consisting of 1 guanine, followed by 6 CTGs, complementary to the CAG repeat, were found to specifically reduce mutant htt and ataxin-3 protein levels in patient-derived cells [37], [38]. Although the reduction in protein levels by PNA transfection was highly efficient with very long stretches of CAGs, there was only a minor decrease when the number of CAG repeats that occur most frequently in the patient population was targeted [38], [39]. Testing a variety of modifications resulted in oligonucleotides with a thymine (T) LNA nucleotide at every third base (LNA(T)) and 2′*O*,4′*O*-C-ethyl nucleic acid (cET) which show higher selectivity (2.9 and 3.7 fold) for mutant alleles with 41 CAG repeats [36].

AONs are a promising therapeutic tool, as was recently shown by phase I and phase I/II clinical trials in Duchenne muscular dystrophy (DMD) boys carrying specific deletions in the *DMD* gene [60]. Local and systemic (subcutaneous) delivery of a specific 2′OMe modified AON induced exon 51 skipping in the *DMD* gene on transcript level allowing the synthesis of novel, internally deleted, but likely (semi-) functional, dystrophin proteins without clinically apparent adverse events [61]. AONs have also been used for the treatment of neurodegenerative disorders and are found to be taken up by neurons when delivered into the cerebral lateral ventricles. As treatment for ALS 2′-O-methoxyethyl modified deoxynucleotides infused intraventricularly were found to reduce both SOD1 transcript and protein levels in rats and rhesus monkeys, which resulted in a slower disease progression [34]. Similarly modified oligonucleotides for spinal muscular atrophy (SMA) resulted in putative therapeutic levels in all regions of the spinal cord after intrathecal infusion in non-human primates [62].

The exact mechanism by which the AONs are used in the current study to reduce transcript levels and why they show both an allele and gene preference is not known. This selective repeat-length dependent reduction was also seen in myotonic dystrophy type 1 after (CAG)_7_ AON treatment [63]. Since 2′OMe PS modified AONs are nuclease and RNase H resistant, RNase H-induced cleavage or RISC mediated degradation of dsRNA is not likely to be involved [63]. Another explanation could be RNase-independent translational blocking by (CUG)_7_ AON binding to the transcript, preventing binding or steric blockage of the ribosomal units. However, translational blocking is not likely to be involved since htt transcript levels are also reduced [38]. Reduction of transcript levels are not thought to be caused by interference of the (CUG)_7_ AON during cDNA synthesis. Addition of (CUG)_7_ AON just prior to the mRNA before cDNA synthesis did not result in reduced htt transcript levels (data not shown). A more likely explanation for the allele specific effect of the (CUG)_7_ AON shown in the current paper could be caused by structural differences in transcripts with normal and expanded repeats. Expanded CAG repeats are known to from hairpin structures [64]. (CUG)_7_ AON binding could stabilize this CAG RNA hairpin, resulting in selective breakdown of the mutant transcripts. Another explanation could be that the expanded CAG repeats have a more open structure, making them more accessible for AON binding, thereby leading to induction of selective breakdown, resulting in a lower mRNA expression. These two models are not mutually exclusive and other mechanisms may as well be involved.

However, these results show that reduction of the mutant mRNA and/or its translation are promising generic routes towards therapy of triplet expansion diseases. Our future plans would be unraveling the exact mechanism of the reduction of HTT transcripts by the AON and *in vivo* testing of the toxicity and delivery of the (CUG)_7_ in animal models of polyQ diseases.

Here we show the first evidence of a specific reduction of mutant htt, ataxin-1 and -3, and atrophin-1 transcript levels using 2′OMe PS modified AONs that recognizes pure CAG repeat stretches, suggesting that a single AON is potentially applicable to polyQ neurodegenerative diseases with an expanded pure CAG repeat.

## Materials and Methods

### Cell culture and Transfection

Patient derived fibroblasts from HD (GM04022), SCA3 (GM06151), SCA1 (GM06927), and DRPLA (GM13716) (purchased from Coriell Cell Repositories, Camden, USA); and control fibroblasts FLB73 (kind gift from Dr. M.P.G. Vreeswijk, LUMC) were cultured at 37°C and 5% CO_2_ in Minimal Essential Medium (MEM) (Gibco Invitrogen, Carlsbad, USA) with 15% heat inactivated Fetal Bovine Serum (FBS) (Clontech, Palo Alto USA), 1% Glutamax (Gibco) and 100 U/ml penicillin/streptomycin (P/S) (Gibco). Human Epstein Barr Virus transformed lymphoblasts HL2.42 and HL2.93 were a kind gift from Prof. E. Bakker (Laboratory of Diagnostic Genome Analysis (LDGA), LUMC). Cells were cultured at 37°C and 5% CO_2_ in RPMI 1640 medium (Gibco), containing 15% FBS, 1% glutamax and 100 U/ml P/S.

AON transfection was performed with 3.3 µl ExGen 500 polyethylenimine (PEI) (MBI Fermentas, Vilnius, Lithuania) per µg AON. AON and PEI were diluted in 150 mM NaCl to a total volume of 100 µl and mixtures were prepared according to the manufacturer's instruction. Four different transfection conditions were used: 1) transfection with 1–100 nM (CUG)_7_, 100 nM (CUG)_3_, 100 nM (CUG)_12_, 2) transfection with 10–100 nM h40AON2 directed against exon 40 of the *DMD* gene (5′- UCC UUU CAU CUC UGG GCU C -3′) (Control AON) [65], 3) transfection without AON (Mock II), and 4) NaCl only (Mock I). Mixtures were added to a total volume of 2 ml of medium with 5% FBS. Four hours after transfection, medium was replaced with fresh medium and a second identical transfection was performed 24 hours after the first transfection. All AONs consist of 2′-O-methyl RNA and contain a full-length phosphorothioate backbone (Prosensa B.V. Leiden, the Netherlands).

### RNA Isolation and RT-PCR

Forty eight hours after the first transfection cells were harvested by trypsinization and washed twice with Hanks buffered salt solution (HBSS) (Gibco). Total RNA was isolated from the cells using an RNeasy Mini Kit (QIAgen, Venlo, The Netherlands), with an on-column DNase treatment for approximately 30 minutes. RNA was eluted in 50 µl elution buffer and cDNA was synthesized from total RNA using the Transcriptor First Strand cDNA Synthesis Kit with Random Hexamer primers at 65°C (Roche, Mannheim, Germany).

PCR was performed using 1 µl cDNA, 10x PCR buffer with 1.5M MgCl_2_ (Roche), 2 mM dNTPs, 10 pmol forward primer, 10 pmol reverse primer, 1U FastStart Taq DNA Polymerase (Roche), 1 M Betaine (Sigma-Aldrich, St. Louis, USA), and PCR grade water to a final volume of 20 µl. PCR was performed with primers for HTT, ATXN1, ATXN3, and ATN1 (all flanking the CAG repeat), ACTB, and RPL22 (for sequences, see Table S1). The PCR program started with a 4 min initial denaturation at 95°C, followed by 35 cycles of 30 sec denaturation at 95°C, 30 sec annealing at 60°C (56°C for ATXN3), 45 sec elongation at 72°C, after which a final elongation step was performed at 72°C for 7 min.

Lab-on-a-Chip was performed on the Agilent 2100 Bioanalyzer (Agilent Technologies, Waldbronn, Germany), using the Agilent DNA 1000 Kit. Expression levels were normalized for β-actin levels and relative to transcript levels without transfection (Mock I). The relative mutant transcript levels were analyzed using a paired two-sided Student *t* test. Differences were considered significant when *P*<0.05.

### qPCR, Calculations and Sequencing

The qPCR was performed using 1 µl of 5x diluted cDNA, 2x FastStart Universal SYBR Green Master mix (Roche), 2.5 pmol forward primer, 2.5 pmol reverse primer and PCR grade water to a total volume of 10 µl. Primer pairs for 6 transcripts containing long uninterrupted CAG repeats were selected for qPCR by BLAST analysis and ACTB and RPL22 were used as reference genes. (For primer list, see Table S1). The qPCR was performed using the LightCycler 480 (Roche). Initial denaturation was 10 min. at 95°C, followed by 45 cycles of 10 sec. denaturation at 95°C, 30 sec. annealing at 59°C and 20 sec. elongation at 72°C. The final elongation was performed 5 min. at 72°C. Next, we performed a melting curve analysis of all samples from 60°C to 98°C with a ramp rate of 0.02°C per sec.

Relative expression of the transcript levels was calculated as described previously [66]. All samples were run in triplicate on a plate and two independent experiments were performed for each sample. On all plates both reference genes were included to correct for inter-plate variance.

Primer efficiencies were determined using LinRegPCR v11.1[67] with the raw data amplification curves as input and Mock II was used as reference. Values from the mock water transfected cells (Mock I) were set on 100%. The relative transcript levels were analyzed using a paired two-sided Student *t* test. Differences between groups were considered significant when *P*<0.05.

CAG repeats of the CAG enclosing transcripts were amplified using primers flanking the CAG repeat (see Table S1). PCR products were loaded on an agarose gel and bands were extracted using the QIAquick Gel Extraction Kit (QIAgen). The purified products were sequenced by Sanger sequencing, using the Applied Biosystems 96-capillary 3730XL system (Life Technologies Corporation, Carlsbad, USA) with the Applied Biosystems BigDyeTerminator v3.1 kit.

### Protein isolation and Western blotting

Cells were detached from the culture surface with a 0.5% Trypsin/EDTA solution. After washing twice with 1x HBSS, cells were resuspended in 200 µl ice cold lysis buffer, containing 1x PBS, 0.4% Triton-X100, and 1 tablet Complete mini protease inhibitor EDTA free (Roche) per 10 ml buffer. Next, samples were sonicated 3 times for 5 seconds using ultrasound with an amplitude of 60 at 4°C. After incubation in a head-over-head rotor at 4°C for 1 hour, the extract was centrifuged for 15 min at 10,000*g* and 4°C and supernatant was isolated. Protein concentrations were determined by the bicinchoninic acid kit (Thermo Fisher Scientific, Waltham, USA) using Bovine Serum Albumin as a standard. Samples were snap frozen and stored at −80°C.

Protein extracts were separated by SDS-PAGE, with 4–15% acryl/bisacrylamide 1∶37.5 separating gels and 30 µg (human fibroblasts) of protein lysate loaded. For each sample the Spectra Multicolor High Range Protein Ladder (Fermentas) was used as a marker. Electrophoresis was performed for 30 min at 100V through the stacking gel and 5 hours at 150V through the running gel. Gels were blotted onto a polyvinylidene fluoride (PVDF) membrane for 3 hours at 300 mA. Membranes were blocked with 1x Tris Buffered Saline +0.5% Tween 20 (TBST) containing 5% non-fat milk powder (Profitar Plus, Nutricia, Zoetermeer, the Netherlands). The antibodies used for detection were mouse 4C8 for htt (Eurogentec, Liege, Belgium) dilution 1∶1000, mouse 1C2 specific for expanded poly glutamine stretches (Eurogentec) dilution 1∶500, mouse ataxin-3 (Eurogentec) 1∶1000, rabbit TBP (Santa Cruz Biotechnology, USA) 1∶1000, and mouse β-actin, diluted 1∶5000. Secondary antibodies were goat α-mouse-horseradish peroxidase (Santa Cruz) and goat α-rabbit-horseradish peroxidase (Santa Cruz), both diluted 1∶10.000 in 1x TBST. Horseradish peroxidase was activated by ECL+ reagent (GE Healthcare, Buckinghamshire, United Kingdom) to visualize positive staining on film.

Protein bands were quantified using ImageJ software. The percentage of inhibition was calculated as a relative value to a non-treated control sample and was normalized using β-actin.

## Supporting Information

Table S1 **Used primers for Sanger sequencing and (quantitative) RT-PCR.** *Abbreviations*: AR, androgen receptor; ATN1, atrophin-1; ATXN1, ataxin-1; ATXN2, ataxin-2; ATXN3, ataxin-3; GLS, glutaminase; HTT, huntingtin; TBP, TATA box binding protein; ZNF384, zinc finger protein 384; ACTB, β-actin; RPL22: ribosomal protein L22.
(DOC)
