# Supplementary material for: Targeting Several CAG Expansion Diseases by a Single Antisense Oligonucleotide
Source: PLoS One. 2011 Sep 1;6(9):e24308. doi: 10.1371/journal.pone.0024308 (PMC3164722; doi:10.1371/journal.pone.0024308)
Supplement: Table S1 — Used primers for Sanger sequencing and (quantitative) RT-PCR. Abbreviations: AR, androgen receptor; ATN1, atrophin-1; ATXN1, ataxin-1; ATXN2, ataxin-2; ATXN3, ataxin-3; GLS, glutaminase; HTT, huntingtin; TBP, TATA box binding protein; ZNF384, zinc finger protein 384; ACTB, β-actin; RPL22: ribosomal protein L22. (DOC) [file pone.0024308.s001.doc]

| **Transcript** | **Direction** | **Amplifying the CAG repeat** | **Without CAG repeat** |
| --- | --- | --- | --- |
| HTT | Forward | ATGGCGACCCTGGAAAAGCTGAT | ATGGCGACCCTGGAAAAG |
|  | Reverse | TGAGGCAGCAGCGGCTG | CTGCTGCTGGAAGGACTTG |
| AR | Forward | GACCTACCGAGGAGCTTTCC | TGCAACTCCTTCAGCAACAG |
|  | Reverse | CTCATCCAGGACCAGGTAGC | TCGAAGTGCCCCCTAAGTAA |
| ATXN1 | Forward | TGGAGGCCTATTCCACTCTG |  |
|  | Reverse | TGGACGTACTGGTTCTGCTG |  |
| ATXN2 | Forward | CCTCACCATGTCGCTGAAG | CTCCGCCTCAGACTGTTTTG |
|  | Reverse | GGAGACCGAGGACGAGGAC | GAGAAGGAGGACGACGAAGG |
| ATXN3 | Forward | GAGCTTCGGAAGAGACGAGA | GGGGACCTATCAGGACAGAG |
|  | Reverse | GATCACTCCCAAGTGCTCCT | CAAGTGCTCCTGAACTGGTG |
| ATN1 | Forward | CACCCACCAGTCTCAACACA | TCACAGCCAGGTGTCCTACA |
|  | Reverse | GAGACATGGCGTAAGGGTGT | GTAGCCGAAGAGGTGGTGAC |
| GLS | Forward | TAGGCGGAGCGAAGAGAAC | ACCCAAGTAGCTGCCCTTTC |
|  | Reverse | GCTCAACAGGGGAGGATG | GCTCAACAGGGGAGGATG |
| TBP | Forward | GACCCCACAGCCTATTCAGA | CCACAGCTCTTCCACTCACA |
|  | Reverse | TTGACTGCTGAACGGCTGCA | GCGGTACAATCCCAGAACTC |
| ZNF384 | Forward | ACATATGCGCAAACACAACC | CCACCACACTTCCAGTCTCC |
|  | Reverse | CCAGGAGACTGGAAGTGTGG | TGACAGTGAGGCAGATGTCC |
| ACTB | Forward |  | GGACTTCGAGCAAGAGATGG |
|  | Reverse |  | AGCACTGTGTTGGCGTACAG |
| RPL22 | Forward |  | TCGCTCACCTCCCTTTCTAA |
|  | Reverse |  | TCACGGTGATCTTGCTCTTG |
